# Sharing Different Reference Frames: How Stimulus Setup and Task Setup Shape Egocentric and Allocentric Simon Effects

**DOI:** 10.3389/fpsyg.2018.02063

**Published:** 2018-11-30

**Authors:** Pamela Baess, Tom Weber, Christina Bermeitinger

**Affiliations:** Institute of Psychology, University of Hildesheim, Hildesheim, Germany

**Keywords:** egocentric frame of reference, allocentric frame of reference, Simon effect, task sharing, joint action

## Abstract

Different reference frames are used in daily life in order to structure the environment. The two-choice Simon task setting has been used to investigate how task-irrelevant spatial information influences human cognitive control. In recent studies, a Go/NoGo Simon task setting was used in order to divide the Simon task between a pair of participants. Yet, not only a human co-actor, but also even an attention-grabbing object can provide sufficient reference in order to reintroduce a Simon effect (SE) indicating cognitive conflict in Go/NoGo task settings. Interestingly, the SE could only occur when a reference point outside of the stimulus setup was available. The current studies exploited the dependency between different spatial reference frames (egocentric and allocentric) offered by the stimulus setup itself and the task setup (individual vs. joint Go/NoGot task setting). Two studies (Experiments 1 and 2) were carried out along with a human co-actor. Experiment 3 used an attention-grabbing object instead. The egocentric and allocentric SEs triggered by different features of the stimulus setup (global vs. local) were modulated by the task setup. When interacting with a human co-actor, an egocentric SE was found for global features of the stimulus setup (i.e., stimulus position on the screen). In contrast, an allocentric SE was yielded in the individual task setup illustrating the relevance of more local features of the stimulus setup (i.e., the manikin’s ball position). Results point toward salience shifts between different spatial reference frames depending on the nature of the task setup.

## Introduction

Imagine yourself as the pilot in a cockpit of an airplane. In front of you, there is a multitude of displays, electronic flight instruments, and instruments ensuring the safety during your flight. Next to you is another pilot who – alongside with you – controls and checks all visual aids for flight security. As far as operational issues, both pilots share the responsibility it takes to manage the flight and incoming stream of information provided by all visual displays. In general, human-machine displays are an example par excellence for demonstrating the requirement of forming spatial codes in order to structure the environmental input. One dominant way of structuring the environment makes use of spatial labels such as up and down or left and right. With reference to the example of the cockpit, flying is a shared responsibility involving both pilots and still requires the formation of one’s own spatial codes while concurrently representing the task and responsibilities of the other pilot as well.

In laboratory tasks, it is well-known that the information regarding a spatial location of a stimulus is hard to ignore, even though completely task-irrelevant, which became known as the Simon effect (SE) (for review, Simon, 1990; Lu and Proctor, 1995; Proctor and Vu, 2006; Hommel, 2011). In a Simon two-choice task setting, participants have to respond to one stimulus feature (for example, red and green color) through assigning one response to each color.

For example, the left/right response button is required as a reaction to a green/red stimulus shown on the screen if the left/right button was assigned to green/red through means of task instruction. The location of the stimuli varied displayed either on the left or right side of the screen’s center (e.g., Craft and Simon, 1970). Interestingly, responses were much quicker when stimulus location and response location overlap [stimulus–response (SR) compatible], saying green stimulus on the left side requiring the left button response than when they do not overlap (SR incompatible). This difference in reaction time is referred to as the SE and it is explained in terms of an interaction between two parallel and independent processing routes connecting perception to action: an unconditional and a conditional component (Kornblum et al., 1990; Dejong et al., 1994). The unconditional route leads to automatic activation of a spatially corresponding response (for example, stimulus on left side triggering left response), irrespective of task instructions. Contrary, in the conditional route, the response is activated based on the task-required associations between stimuli and spatial codes (for example, left button when green stimulus occurred). Importantly, the effects of both routes overlap for SR compatible trials (e.g., green stimulus on left side requiring left response). Hence, in case of SR incompatible trials (e.g., green stimulus on right side requiring left response), both activated responses differ. Here, a conflict between both activations is the result causing a slowdown of response speed.

If one response alternative is removed (and thus no source of conflict between stimulus codes and response codes available, codes referring to the cognitive representation of stimulus and response, respectively), rendering the task from a two-choice task setting to a Go/NoGo task setting (e.g., react only to green stimuli with the left button and withdraw from responding for red stimuli), typically no reliable SEs are obtained (Hommel, 1996; Shiu and Kornblum, 1999; Ansorge and Wuhr, 2004) which is explained by the absence of the source of response conflict in Go/NoGo task settings.

Most compelling was the seminal finding of Sebanz et al. (2003) reporting the re-occurrence of a SE in a Go/NoGo task setting when sharing the task with a partner in such a way that each participant is responsible for reacting only to a particular stimulus color with a specified response button, but no SE in an individual Go/NoGo task setting. This was further interpreted as a so-called joint SE (JSE), i.e., the SE in the tradition of the two-choice task setting through dividing the Simon task between two participants (for review, Dolk et al., 2014), introducing the idea of a co-representation of the co-actor’s task. Although others (Dolk et al., 2011, 2013; Dittrich et al., 2012, 2013) emphasized that a human co-actor is not necessarily required in order to obtain a JSE (see below), it nevertheless is theoretically fascinating as under certain conditions, a co-actor or object might provide a spatial reference in joint Go/NoGo task settings. Thus, the co-actor or object apparently strengthened a spatial representation of the task (e.g., I-Go, You-Go) in order to reintroduce response conflict as the source for the reoccurrence of a SE. Here, we will refer to “*task setting*” in order to differentiate between two-choice and Go/NoGo variants of the Simon task. Different versions of Go/NoGo task settings were contrasted: with the presence of a co-actor (joint Go/NoGo task setting) or alone (individual Go/NoGo task setting). Both Go/NoGo task settings introduce variations in the *task setup:* a joint task setup required the differentiation between the responding agents (I-Go vs. You-Go), and thus cognitive representations as the basis of this differentiation, however this was not required in the individual task setup. Thus, the task setup might include cognitive representations of how the stimuli and responses in the Go/NoGo task setting were divided between two participants. In the joint task setup, this includes how both participants represent their part of the Simon task, including their critical stimulus feature (e.g., green or red) and response button (left or right button). Contrary, in the individual task setup, only cognitive representations of one’s own stimulus feature (e.g., green) and response button are required. In this reading, task setup contains all the representations involved representing one’s own task during a joint or individual Go/NoGo task setting, but alongside with it, even all the other, task-irrelevant specifications how the Go/NoGo task settings are carried out (e.g., physical distance between co-actors; objects in the room). Others have coined the term of “task shaping” (Prinz, 2015; Dolk and Prinz, 2016) in joint two-choice or Go/NoGo task settings as a broader term when studying task setups in the joint or individual context. However, task setup in our reading refers more to the concrete situation in which the two-choice or Go/NoGo task setting is accomplished. In addition, the *stimulus setup* (see below) contains the exact representation of the alignment of the stimuli visible in the Simon task on the screen.

Coming back to the seminal findings of a JSE in joint Go/NoGo task setting, further studies explored how the JSE could be re-established even without a human co-actor (Dolk et al., 2011, 2013; Dittrich et al., 2012, 2013). By enriching the task setup (e.g., by presenting external, attention-grabbing objects or ambiguous response devices), the enriched task setup provided sufficiently salient reference points granting the response conflict as an essential source for the existence of a SE in Go/NoGo task setting. For example, Dolk et al. (2013) documented a SE for an auditory Simon task in the individual Go/NoGo task setting when the task setup included a Japanese waving cat or other attention-grabbing objects placed, for example, on the left side of the participant. Or, even an enriched task setup through increasing the salience of the responses through a joystick (Dittrich et al., 2012) can provide sufficient reference points for the finding of a SE in the individual Go/NoGo task setting. Reports of a SE even without the involvement of a co-actor have promoted the idea of alternative accounts (Dittrich et al., 2012; Dolk et al., 2013) emphasizing the potential role of the cues in the enriched task setup (e.g., provided by an attention-grabbing object) serving as reference points for a spatial coding of the scenario. Importantly, all this research inspected how changes in the task setup can reintroduce a SE with Go/NoGo task setting. However, the possibility that the stimulus setup itself could introduce response conflict in joint and individual Go/NoGo task settings is fairly untested. The current study explicitly explores the role of an enriched stimulus setup by contrasting different task setups, i.e., joint and individual task setups in Go/NoGo task settings. The use of an enriched stimulus setup is based on the assumption to form different spatial reference frames besides the commonly used one with reference to the spatial side regarding the screen’s center.

While the idea of multiple reference frames has received a substantial amount of consideration in studies with the two-choice Simon task settings (for review, Rubichi et al., 2006), it has (almost) been completely neglected in Go/NoGo Simon task settings. In the following, we will first briefly review the available evidence of multiple spatial codes in two-choice task settings and then elaborate about the idea of multiple spatial codes under Go/NoGo task settings.

### Multiple Spatial Codes in Two-Choice Simon Task Settings

As discussed above, the standard, two-choice Simon task setting (for review, Simon, 1990) is explained with the concept of SR overlap. However, the standard stimuli (e.g., green/red circle in the left/right side of the screen’s center) used in Simon tasks offer only one kind of crucial SR-overlap, namely with regard of the center of the screen (which is in this case identical with the participant’s body midline). This is a very reduced labor situation and can by far not be compared to such a complex scenario as given in the cockpit with multiple spatially aligned displays. Therefore, prior research studied whether different SEs indicating the existence of different spatial codes can be reported for the two-choice Simon task setting when the stimulus setup itself provided more reference points for a potential SR-mapping (for review, Rubichi et al., 2006). Crucially, the stimulus setup (and not the task setup) was enriched in order to provide reference points for a spatial coding along the center of the screen (also called hemispace) alongside with a spatial code within the left right side of the screen’s half (labeled as hemifield or relative stimulus position). With reference to the literature, two principle ways of implementation can be contrasted. Some studies used some sort of an “external-object-approach” meaning that the stimulus setup was enriched by presenting additional, external objects (such as vertical lines or horizontally aligned boxes) on the screen in spatial relation to the critical stimulus. These objects however were not part of the critical stimulus as such, but helped to introduce different spatial locations on the screen. Consequently, the critical stimuli could occur on different spatial locations along the horizontal or vertical dimension (Nicoletti and Umilta, 1984; Umilta and Liotti, 1987; Lamberts et al., 1992; Roswarski and Proctor, 1996). For example, in the study by Roswarski and Proctor (1996), three short vertical lines were presented on the screen demarking four potential locations for the occurrence of the critical stimulus (two in each hemifield). Here, SEs occurred for both possible reference frames, i.e., for hemispace (with reference to the center of the screen) and hemifield (referring to the relative position within each hemispace), provided that the reference lines were visible before the critical stimulus. Through such external objects, multiple spatial locations of stimulus occurrence were established allowing the formation of spatial codes, as indicated by the presence of different SEs with regard to different spatial reference frames. Yet, these spatial codes were not formed automatically as the spatial codes, possibly formed for the relative position within each hemispace, might have been overwritten during response selection when the reference frame, and the target stimuli were simultaneously presented. To summarize, the “external object-approach” provided evidence for SE (and/or the SR prober as utilized in some studies) recruiting different spatial reference frames depending crucially on the experimental manipulation: different spatial reference frames were only established when reference objects or spatial cues (for example, indicating the side of the screen of the upcoming stimuli) were provided before the occurrence of the crucial stimulus (Lamberts et al., 1992; Roswarski and Proctor, 1996). In other words, different spatial reference frames (as indicated by SEs) were only formed when additional cues, be it temporal and/ or spatial, were provided. Besides this, a dominant SE based on the center of the screen (i.e., hemispace) was the robust finding.

Another set of studies followed a different procedure (“same-object-approach”) by enriching the stimulus setup through embedding the critical stimulus into a more global object (Wang et al., 2016; Baess and Bermeitinger, unpublished). For example, Wang et al. (2016) presented the critical stimulus, a fork, in combination with another object, here a plate, so that the fork was superimposed on the plate. Participants were required to react to the color of the fork, yet the position of the fork with respect to the plate was completely task-irrelevant. The fork’s position could be assessed in two different ways, regarding one’s own body midline (i.e., egocentric position; fork and plate on the left or right side of the screen’s center) or regarding its position on the plate (i.e., allocentric position; fork on the left or right side of the plate). With this stimulus setup, egocentric and allocentric SEs were simultaneously obtained, however, the allocentric SE was subject to carry-over effects from a preceding spatial judgment task inducing the allocentric perspective. In contrast, Baess and Bermeitinger (unpublished) reported evidence for the simultaneous formation of egocentric and allocentric SEs independent of previous task instructions. The authors used drawings of stick-figure manikins holding a colored ball in either hand (allocentric reference frame). The manikins were presented either at the left or right side of the screen (egocentric reference frame). Here, reliable egocentric (with reference to manikin’s screen position) and allocentric (with reference to manikin’s ball position) SEs simultaneously occurred, without any previous task demands and prior spatial or temporal cues presented before the critical stimulus. A further manipulation contrasted the amount of manikin stimuli (one manikin vs. nine manikins) simultaneously shown on the screen introducing the possibility of another non-spatial perceptional reference frame recruiting the Gestalt law of grouping (Koffka, 1935/1963). Interestingly, the egocentric reference frame interacted with this non-spatial perceptional one: larger egocentric SEs were reliably observed when one manikin was presented compared to when nine manikins were presented simultaneously. In contrast, the allocentric SEs remained unaffected by the manipulation of the non-spatial perceptual reference frame: reliable allocentric SEs were observed for both variants of the non-spatial perceptual reference frame. To conclude, in contrast to the previous external-object-approach, the reference points required for the formation of spatial codes surrounded the critical stimulus itself embedding it into another, more global object. Throughout the rest of this paper, we will use “egocentric” SEs when referring to the reference frame based on the screen’s center (in the “external-object approach” labeled as hemispace) and “allocentric” SEs when the reference frame was given as part of a more global object (in the “external-object-approach,” called as hemifield).

### The Present Study

Although the idea of different spatial reference frames present in one stimulus setup is well-rooted, not much is known how different spatial reference frames are shared between two participants. As shown with the case of the JSE (for review, Dolk et al., 2014), a co-actor or an attention-grabbing external object have proven to be salient enough to enrich the task setup in order to provide a reference frame (and thus a JSE) even in joint and individual Go/NoGo task settings. The possibility of an interaction between reference frames introduced by the task setup and those implemented through the stimulus setup is definitely fascinating. To the best of our knowledge, only the study by Ciardo et al. (2016) addressed this question so far. Using the “external-object-approach” by dividing the screen with short vertical lines, the critical stimulus could occur randomly at any of the four different locations. Thus, the stimulus setup included two different reference frames, namely hemispace (in our reading, egocentric) and hemifield (in our reading, allocentric). Moreover, the task was conducted either together with a co-actor or alone (joint and individual Go/NoGo task setting). Evidence was reported for a SE for hemispace in the joint Go/NoGo task setting, but not in the individual Go/NoGo task setting. No SEs based on hemifield in either task setup were observed. In a further experiment, two participants performed the task as two-choice task setting with this stimulus setup under joint and individual task setup. Here, SEs for both spatial reference frames were observed and no difference based on task setup was evident. Consequently, this study provides initial evidence for the interaction between different spatial reference frames provided by stimulus setup and task setup. Yet, to state, it is unclear how different spatial reference frames given through task setup and stimulus setup are effective when using the “same-object-approach” with an enriched stimulus setup. Therefore, the present research investigated the formation of different spatial reference frames (as indexed by the egocentric and allocentric SEs) – as indicative of an enriched stimulus setup – under different, i.e., individual and joint, task setups with Go/NoGo task settings further. The present study was tailored to investigate the formation of multiple spatial codes in joint and individual Go/NoGo task settings using the enriched stimulus setup with stick-figure manikins.

Of particular interest to us is how different task setups (individual vs. joint) influence the formation of multiple reference frames based on the stimulus setup presented. Based on the previous literature with enriched task setup, i.e., individual and joint, respectively, with Go/NoGo task settings, one would predict a SE in the joint Go/NoGo task setting, but none in the individual one: an egocentric SE based on the stimulus’ screen position should be only elicited when a co-actor or an attention-grabbing object provides sufficient reference as a source for the occurrence of cognitive conflict. Regarding the other, allocentric SE based on the manikin’s relative ball position, the hypothesis would be similar: no SE in the individual Go/NoGo task setting and if one, then a SE in the joint Go/NoGo task setting. Alternatively, the available evidence of different spatial reference frames in enriched stimulus setups points toward the possibility of simultaneous egocentric and allocentric SEs in the two-choice Simon task setting (Baess and Bermeitinger, unpublished). Yet, it is so far unclear whether these two SEs can also be observed in Go/NoGo task settings of this Simon task, albeit in general, SEs are, if not triggered by the task setup, absent in Go/NoGo task settings given the lack of response conflict.

In order to scrutinize the saliency of different reference frames depending on task setup and stimulus setup, we conducted three experiments. Experiment 1 used the stick-figure manikins of the two-choice version of the Simon task with egocentric and allocentric reference frames (Baess and Bermeitinger, unpublished) under an individual and joint Go/NoGo task setting. Experiment 2 repeated Experiment 1 with different stimulus material. In Experiment 3, an external, attention-grabbing object (i.e., a Japanese waving cat) was placed next to the participant using otherwise the same stimulus setup as in Experiment 1. Across all three experiments, the enriched stimulus setup with different spatial reference frames remained constant, but the task setup changed (with/without co-actor/attention-grabbing object).

## Experiment 1

The present experiment implemented a Go/NoGo version of the two-choice egocentric and allocentric Simon task introduced by Baess and Bermeitinger (unpublished). Drawings of stick-figure manikins with a ball of blue or yellow color in either hand (allocentric reference frame) were presented left or right to the screen’s center (egocentric reference frame). With this enriched stimulus setup, both spatial reference frames were instantly processed as indicated by reliable egocentric and allocentric SEs in the two-choice task setting. Further, Baess and Bermeitinger (unpublished) reported that the size of the egocentric SE (i.e., based on the manikin’s position with reference to the screen’s center) was modulated by a non-spatial perceptual reference frame as introduced through the amount of identical stimuli shown on the screen.

The current experiment used exactly the same version of the Simon task in a Go/NoGo task setting. The task was divided between two participants in such a way that each one was responding only to one stimulus color (i.e., color of the ball in the manikin’s hand: blue or yellow). The task setup contained a joint Go/NoGo task setting and an individual Go/NoGo task setting. Moreover, the amount of stimuli presented on the screen was manipulated: either one manikin or a set of nine manikins was simultaneously shown. This variation created the possibility of another non-spatial perceptual reference frame based on the Gestalt law of grouping (Koffka, 1935/1963). As this non-spatial perceptual reference frame influenced particularly the size of the egocentric SE in the two-choice task setting (Baess and Bermeitinger, unpublished), it was also included in the present set of studies.

Based on previous literature on the influence of task setup on the emergence of a SE (for review, Dolk et al., 2014), egocentric and allocentric SEs should occur in the joint Go/NoGo task setting, but not the individual one. As suggested by the two-choice task setting (Baess and Bermeitinger, unpublished), when nine manikins were simultaneously shown on the screen, responses should be generally faster than when one manikin was presented.

### Materials and Methods

#### Subjects

Forty participants were recruited for this study. One participant was excluded due to a lack of compliance (two-choice responses in single Go/NoGo condition). Thus, the final sample consisted of 39 participants (mean age: 21.6 years; 19–34 years, five male). Six participants were left-handed (mean laterality quotient: -55.80, *SD* = 46.30) as assessed with a handedness questionnaire (Oldfield, 1971). Participants were individually recruited through advertisement at the University of Hildesheim and received partial course credit for participation. Parts of the experiments were performed together with a same gender participant. Their personal relationship was assessed with the IOS scale (Aron et al., 1992) showing a mean relationship of 2.64 (1.48 *SD*) on a scale from 1 to 7. All participants gave written informed consent and were treated in accordance to the Declaration of Helsinki. The study was approved by the local ethic’s committee of the University of Hildesheim (“Fachbereich 1”).

#### Stimuli and Experimental Tasks

**FIGURE 1:**
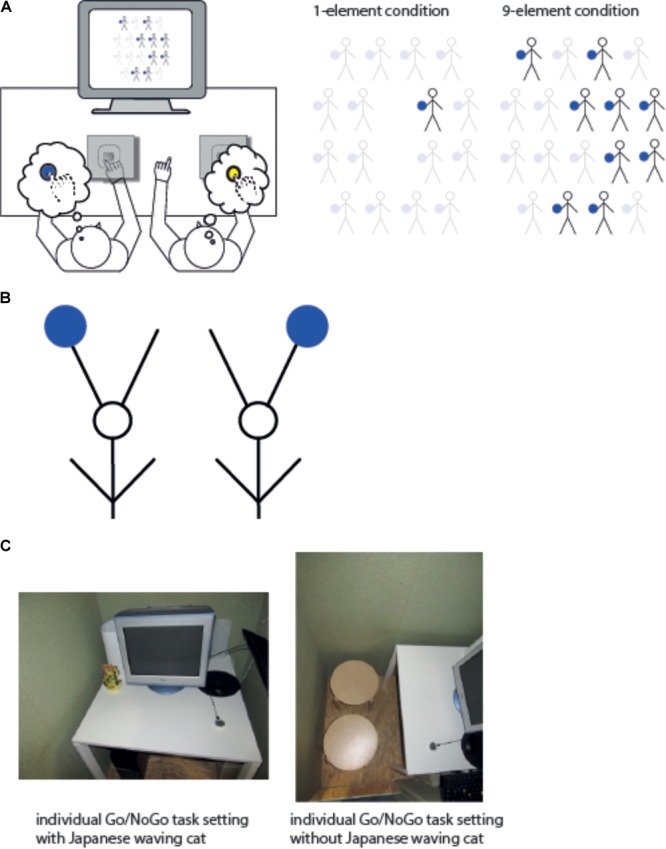
**(A)** Left column: Experimental setup used in Experiments 1 and 2. A variant of the Simon task was split between two participants in such a way that each one responded to one particular stimulus color (here: Participant A to blue stimuli and Participant B to yellow stimuli). The experimental task was carried out under a joint (as shown here) and an individual Go/NoGo Task setting. The seating position and stimulus color assignment to each participant remained the same during the whole experimental session. Right column: Shown is the stimulus layout visible on the screen, separately for the one-element and nine-element condition. In the one-element condition, one stimulus was shown at a time occurring randomly at any out of 16 possible locations centered around the midline of the screen. Nine identical stimuli were shown simultaneously in the nine-element condition (note: due to the use of two midline stimulus locations, the set of nine stimuli occurred pseudo-randomly involving 9 of 18 possible stimulus locations). **(B)** Abstract geometrical patterns used as stimulus material in Experiment 2. **(C)** Task setup for Experiment 3. Participants performed the Go/NoGo task setting with (left part, individual Go/NoGo task setting with Japanese waving cat) or without (right part, individual Go/NoGo task setting without Japanese waving cat) an unrelated Japanese waving cat. Different visual angles in the pictures served only to illustrate the layout of the Experiment 3 better.

Stick-figure manikins holding a blue or yellow ball were used as stimuli (Figure 1A). The manikins were created using Adobe Illustrator, they were 22 mm in width and 37 mm in height. The critical stimulus feature, i.e., the ball, was 7 mm in diameter. The amount of manikins shown simultaneously on the screen was manipulated (see Figure 1A, right part), i.e., resulting in the one-element and nine-element condition for manipulating the non-spatial perceptual reference frame which was implemented through separate blocks. In both conditions, stimuli occurred randomly at any out of 16 stimulus positions (in case of the nine-element condition, two additional stimulus positions were used, resulting in 18 possible positions including two midline positions, see Figure 1A right part) left or right side of the midline. The stimulus positions were chosen along four imagined rows on the screen [row 1: four positions; row 2: five positions (including one midline position that was only used in the nine-element condition); row 3: five positions (including one midline position that was only used in the nine-element condition); row 4: four positions]. In case of the nine-element condition, nine stimulus positions were filled simultaneously at a given trial in such a way that the majority of all presented stimuli was either on the egocentric left or right side of the screen. In both conditions, the exact stimuli positions used varied on a trial-by-trial basis. Both, the one-element and nine-element condition were performed under two different Go/NoGo task settings. In the joint task setup, a pair of participants performed the Go/NoGo task setting together in such a way that one participant was assigned to one particular stimulus color (i.e., blue or yellow) throughout the whole experiment (see Figure 1A, left part). In contrast, during the individual task setup, the participant performed exactly the same Go/NoGo task setting (i.e., same relevant stimulus color and response button) alone without the involvement of a co-agent. The non-spatial perceptual reference frame as indexed by the one-element or nine-element condition was implemented under both task setups with counterbalanced order across the subjects (i.e., half of the subjects started with one-element condition, the other half with nine-element condition). The stimuli and experimental program was identical to the one used for the two-choice task setting (Baess and Bermeitinger, unpublished).

#### Procedure

After arriving in the laboratory, participants were assigned with another participant. The participants were asked to take a seat on one of the two chairs in a custom-made sound attenuated chamber. Spatial labels regarding the assignment of the chairs (e.g., left vs. right chair) were avoided during the whole experiment. Instead, throughout the whole experiment, both participants were referred to either as Participant A or Participant B and their corresponding chairs where labeled like that. The label of “Participant A” or “Participant B” was randomly assigned between both participants, but remained the same during the whole experiment. The chair and thus the spatial seating position regarding the screen remained the same for each participant during all parts of the experiment. The order of the non-spatial perceptual reference frame (i.e., one-element vs. nine-element condition) was counterbalanced across all participants and remained the same for each part of the Go/NoGo task setting.

The participants were instructed to respond as quickly as possible to their relevant stimulus color (i.e., either blue or yellow) by pressing one of two custom-made response devices with their dominant hand (see also Figure 1A, left part for the setup). The custom-made response buttons did not produce any perceivable sound when executing the button. The response devices and therefore the responses of each participant were covered by a paper box in the joint Go/NoGo task setting.

**FIGURE 2:**
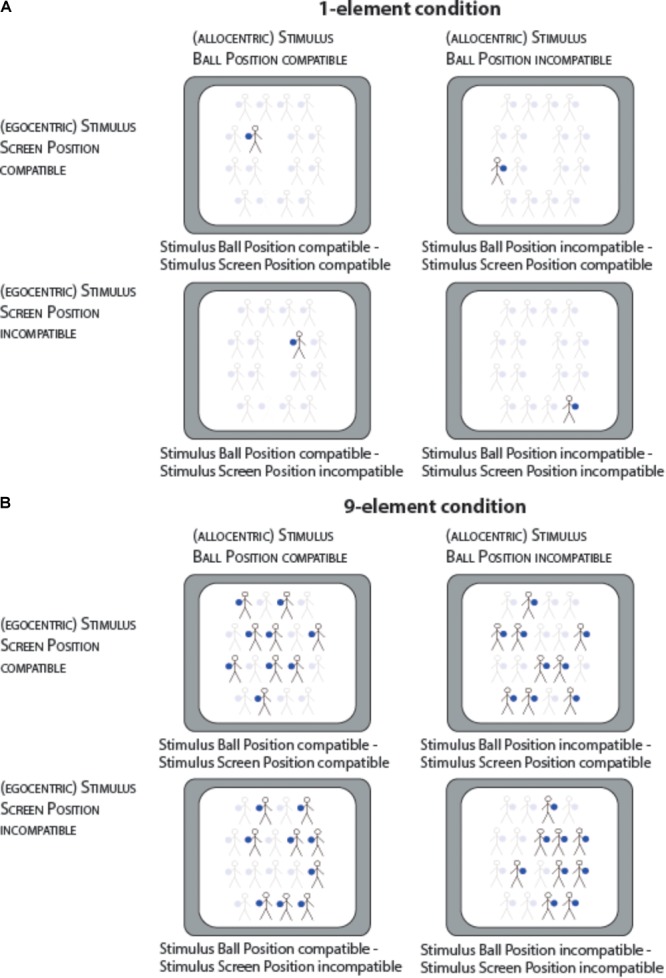
This figure displays all four possible combinations of the experimental factors Stimulus Ball Position (compatible, incompatible) and Stimulus Screen Position (compatible, incompatible) separately for the one-element and nine-element condition. Compatibility labeling refers to the case where a response of the left button is required for a blue stimulus, however, the color-response button associations were alternated across all participants. Transparent stimuli were not visible on the screen and are only displayed for illustrative purpose. In the one-element condition **(A)**, the given stimulus in a trial occurred at any of the 16 lateral positions on the screen (eight left positions, eight right positions). In the nine-element condition **(B)**, 9 of the 18 possible stimuli positions (16 lateral stimulus positions and 2 midline stimulus positions) were filled with the actual stimulus. The majority of the stimuli were on either the left or right side of the screen marking either Stimulus Screen Position compatible or incompatible trials. As shown, the amount of stimuli on the left or right side varied (between 4 and 7, as shown in the examples of the upper and lower panel).

The experiment was run under the Presentation software (Neurobehavioral System, Version 18) on a 16″ color CRT screen (116 cm distance to the participants). For each condition (i.e., one-element or nine-element condition), 192 trials were recorded split into three separate blocks of 64 trials each. The stimuli were shown against a white background for a maximum of 2500 ms or until a response was executed. One trial lasted for max. 4500 ms (500 ms centrally presented fixation cross, max. 2500 ms stimulus duration, 1500 ms inter-trial-interval). In one block, in half of the trials, the (majority of) stimuli were presented on the egocentric left side of the screen and in the other half, the (majority of) stimuli were presented on the egocentrically right side. Orthogonally to this, the ball was for half of the trials on the left side of the manikin and for other half on the right side of the manikin. This ensured that each combination of manikin’s screen position and manikin’s ball position was presented equally often. As shown in Figure 2, four different cases can be differentiated as a function of manikin’s screen position (egocentric reference frame) and manikin’s ball position (allocentric reference frame): (1) Screen Position-compatible – Ball Position-compatible trials, (2) Screen Position-incompatible – Ball Position-compatible trials, (3) Screen Position-compatible – Ball Position-incompatible trials, and (4) Screen Position-incompatible – Ball Position-incompatible trials.

For half the trials in each manikin position × ball position condition, the ball in the manikin’s hand was blue whereas for the other half, the ball was yellow. In total, eight trials were presented per block for each combination of (egocentric) manikin position, (allocentric) ball position, and ball color.

The response of each participant was required in half of the trials of one block (i.e., either for the yellow or the blue balls). For half of the participants, Participant A responded to blue stimuli whereas Participant B reacted to yellow stimuli.

The experiment started with a short training (20 trials in total) together with a partner. At the end of the training block (but not in the other parts of the experiment), the participants received visual feedback regarding the accuracy of the button presses.

After the training, Participant B left the chamber, filled out the IOS scale and handedness questionnaire, and performed another task completely unrelated to the present experiment (in the same room, but outside of the chamber). The other participant (Participant A) executed both versions of the non-spatial perceptual reference frame (i.e., one-element and nine-element condition, counterbalanced order across the participants) under the individual Go/NoGo task setting. After completion, both participants performed both variants of the non-spatial perceptual reference frame (i.e., one-element and nine-element condition, in the same order as the individual Go/NoGo task setting) under the joint Go/NoGo task setting. Finally, the Participant B executed both versions of the non-spatial perceptional reference frame (i.e., one-element and nine-element condition) whereas Participant A filled out the IOS scale and handedness questionnaire and performed another unrelated experiment outside of the chamber. Participant A sat always on the left chair and Participant B on the right chair (distance between both participants: 60 cm), however, the relevant stimulus color was varied between both participants.

#### Data Analysis

Only correct trials were analyzed further (1.07% of all trials were erroneous). Outlying reaction times were identified as 1.5 interquartile ranges above the third quartile with respect to the individual responses times (Tukey, 1977) or below 100 ms. In total, 10.3% of trials were discarded as outliers.

Data were analyzed with a repeated measures analysis of variance (ANOVA) with the within-subject factors Task Setup (individual Go/NoGo, joint Go/NoGo), Number of Elements (one-element condition, nine-element condition), egocentric Stimulus Screen Position (compatible, incompatible to participant’s side), and allocentric Stimulus Ball Position (compatible, incompatible to participant’s side). In addition, additional analysis included Task Order (single Go/NoGo task first, joint Go/NoGo task first) as between-subjects variable in the outlined repeated measures ANOVA. Mean values are given along with standard errors of the mean (SEM).

### Results

**FIGURE 3:**
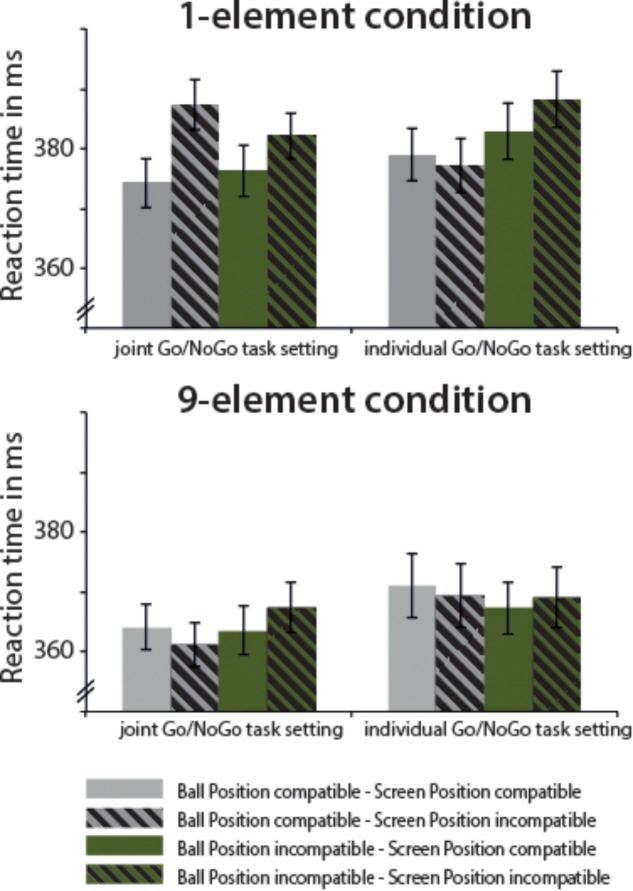
Mean reaction times and SEM for Experiment 1, separated for the one-element and the nine-element condition. Solid bars represent conditions, in which manikin’s position at the screen and participant’s seating position are compatible (i.e., SR compatible), dashed bars display conditions, in which the manikin’s position at the screen and the participant’s seating position are incompatible (i.e., SR incompatible). Gray bars show conditions, in which the ball’s position and the participant’s seating position are compatible, green bars illustrate conditions, in which the ball’s position and the participant’s seating position are incompatible. Bars are given separately for the individual and joint Go/NoGo task setting.

The overall ANOVA yielded a significant effect of Number of Elements, *F*(1,38) = 23.07, MSE = 31,912.27, *p* < 0.001, $\eta_{p}^{2}$ = 0.378, indicating faster responses for the nine-element condition (366.63 ms ± 8.37) compared to the one-element condition (380.93 ms ± 8.20). The main effect of Stimulus Screen Position was almost significant, *F*(1,38) = 3.78, MSE = 1352.01, *p* = 0.059, $\eta_{p}^{2}$ = 0.090, illustrating generally faster responses for egocentrically compatible SR mappings (372.31 ms ± SEM) compared to egocentrically incompatible SR mappings (375.25 ms ± SEM). The interaction between Number of Elements and Stimulus Screen Position was significant, *F*(1,38) = 3.99, MSE = 1110.28, *p* = 0.053, $\eta_{p}^{2}$ = 0.095. We further received an interaction between Number of Elements, Task Setup, and allocentric Stimulus Ball Position, *F*(1,38) = 4.45, MSE = 1913.02, *p* = 0.041, $\eta_{p}^{2}$ = 0.105. The results are displayed in Figure 3. Based on these interactions and following our research interest, we disentangled the interactions by conducting separate analysis for the one-element condition and the nine-element condition. In addition, the analysis including the potential effect of Task Order (single Go/NoGo first, joint Go/NoGo first) showed no main effect of Task Order and importantly, the five-way interaction between Task Order × Number of Element, Task Setup, Stimulus Ball Position, Stimulus Screen Position was clearly not significant (see Appendix Table A1 for the complete summary of the ANOVA).

#### One-Element Condition

The overall ANOVA with the factors Task Setup, egocentric Stimulus Screen Position, and allocentric Stimulus Ball Position revealed a significant main effect of Stimulus Screen Position, *F*(1,38) = 5.91, MSE = 2456.34, *p* = 0.020, $\eta_{p}^{2}$ = 0.135, pointing to faster responses for SR compatible trials (378.12 ms ± 8.30) compared to incompatible ones (383.74 ms ± 8.26). More interestingly, we found two interactions involving the factor Task Setup, i.e., an interaction Task Setup × egocentric Stimulus Screen Position, *F*(1,38) = 5.79, MSE = 1147.18, *p* = 0.021, $\eta_{p}^{2}$ = 0.132, and an interaction Task Setup × allocentric Stimulus Ball Position, *F*(1,38) = 4.36, MSE = 1607.37, *p* = 0.044, $\eta_{p}^{2}$ = 0.103. Thereby the three-way interaction between Task Setup × Stimulus Screen Position × Stimulus Ball Position almost reached the significance level, *F*(1,38) = 3.37, MSE = 1007.38, *p* = 0.074, $\eta_{p}^{2}$ = 0.081. In addition, the Task Order was included as a between-subjects factor into the ANOVA mentioned above. This analysis showed that the factor Stimulus Ball Position significantly interacted with the Task Order, *F*(1,37) = 7.70, MSE = 744.98, *p* = 0.009, $\eta_{p}^{2}$ = 0.172, however, the main effect of Stimulus Ball Position remained non-significant. Stimulus Screen Position was not influenced by Task Order as indicated by the significant main effect of Stimulus Position, *F*(1,37) = 5.82, MSE = 2475.78, *p* = 0.021, $\eta_{p}^{2}$ = 0.136, but no interaction with Task Order. Both interactions with Task setup as observed in the omnibus ANOVA were still significant when controlled for the influence of Task Order.

Further analysis was continued with separate ANOVAs for each level of the factor Task setup.

##### Joint Go/NoGo task setting

In a ANOVA with the factors Stimulus Screen Position and Stimulus Ball Position, only the main effect of Stimulus Screen Position yielded significance, *F*(1,38) = 8.22, MSE = 3480.41, *p* = 0.007, $\eta_{p}^{2}$ = 0.178: faster responses were observed for egocentric SR compatible trials (375.28 ms ± 8.28) compared to SR incompatible trials (384.73 ms ± 7.83). The corresponding SEs, i.e., the reaction time difference between SR incompatible trials and SR compatible trials, are given in Table 1. The additional analysis of the influence of Task Order confirmed this pattern of results: only the main effect of Stimulus Screen Position was significant, *F*(1,38) = 8.00, MSE = 3435.91, *p* = 0.008, $\eta_{p}^{2}$ = 0.178, however, the interaction with Task Order was non-significant. No other main or interaction effect was observed. The corresponding mean values are listed in Appendix Table A2.

**Table 1:** Mean reaction times (in ms) and standard error of the mean (SEM) for compatible and incompatible trials as a function of the mapping between Stimulus Ball Position and Stimulus Screen Position in the joint Go/NoGo Task setup and the individual Go/NoGo Task setup, respectively, as well as the egocentric and allocentric Simon Effects (SE, in ms, SEM in parenthesis), separately for the one-element and the nine-element condition from Experiment 1.

		Joint Go/NoGo	Individual Go/NoGo
		Task setting	Task setting
One-element condition	Stimulus Ball Position compatible – Stimulus Screen Position compatible	374.26 (±8.30)	378.99 (±8.70)
	Stimulus Ball Position compatible – Stimulus Screen Position incompatible	387.33 (±8.35)	377.20 (±9.07)
	Stimulus Ball Position incompatible – Stimulus Screen Position compatible	376.31 (±8.73)	382.94 (±9.53)
	Stimulus Ball Position incompatible – Stimulus Screen Position incompatible	382.13 (±7.69)	388.28 (±9.53)
	Egocentric SE (i.e., referring to Stimulus Screen Position)	9.45 (±3.30)*	1.78 (±2.21)
	Allocentric SE (i.e., referring to Ball Position)	–1.57 (±2.67)	7.51 (±3.01)*
Nine-element condition	Stimulus Ball Position compatible – Stimulus Screen Position compatible	364.04 (±7.57)	371.07 (±10.82)
	Stimulus Ball Position compatible – Stimulus Screen Position incompatible	361.14 (±7.26)	369.43 (±10.70)
	Stimulus Ball Position incompatible – Stimulus Screen Position compatible	363.55 (±8.19)	367.30 (±8.76)
	Stimulus Ball Position incompatible – Stimulus Screen Position incompatible	367.39 (±8.35)	369.10 (±10.23)
	Egocentric SE (i.e., referring to Stimulus Screen Position)	0.47 (±2.26)	0.08 (±2.77)
	Allocentric SE (i.e., referring to Ball Position)	2.88 (±2.23)	–2.05 (±3.11)

*Egocentric Stimulus Screen Position and allocentric Stimulus Ball SEs (i.e., the difference between SR incompatible mappings and SR compatible mappings) are presented separately for the one-element and nine-element condition and the joint and individual Go/NoGo Task setting in Experiment 1. Asterisks refer to significant SEs (*p* < 0.05).*

##### Individual Go/NoGo task setting

The ANOVA with the factors Stimulus Screen Position and Stimulus Ball Position yielded a significant main effect of Stimulus Ball Position, *F*(1,38) = 6.22, MSE = 2199.90, *p* = 0.017, $\eta_{p}^{2}$ = 0.141, illustrating faster responses for allocentric SR compatible trials (378.10 ms ± 8.79) compared to incompatible ones (385.61 ms ± 9.38). The additional ANOVA with Task Order obtained a main effect of Stimulus Ball Position *F*(1,37) = 7.99, MSE = 2324.88, *p* = 0.008, $\eta_{p}^{2}$ = 0.178, and a significant interaction of Stimulus Ball Position and Task Order, *F*(1,37) = 9.24, MSE = 2687.89, *p* = 0.004, $\eta_{p}^{2}$ = 0.200 (see also Appendix Table A2). The interaction between Task Order and Stimulus Screen Position was close to significance, *F*(1,37) = 3.72, MSE = 659.91, *p* = 0.062, $\eta_{p}^{2}$ = 0.091, but there was no main effect of Stimulus Screen Position.

#### Nine-Element Condition

The overall ANOVA with the factors Task setup, Stimulus Screen Position, and Stimulus Ball Position revealed no influence of any main factor on the reaction times. There was only a tendency for an interaction between Stimulus Screen Position and Stimulus Ball Position, *F*(1,38) = 3.16, MSE = 505.58, *p* = 0.083, $\eta_{p}^{2}$ = 0.083, but given its tentative nature, it was not analyzed further. Nevertheless, the corresponding SEs are displayed in Table 1, despite failing to reach the significance level. The additional ANOVA with Task Order did not obtain any significant main effect or interaction.

### Discussion

In line with the two-choice task setting with the same stimulus setup, i.e., stick-figure manikins (Baess and Bermeitinger, unpublished), a set of nine manikins was processed faster than a single manikin providing evidence for a non-spatial perceptual reference frame based on the simultaneous presentation of nine manikins centered around the screen’s center, yet still with a spatial alignment in order to allow a spatial left or right coding in a given trial. Further studies are needed in order to address whether the non-spatial reference frame with the nine manikins could also be reported if the nine manikins were exclusively assigned to one side of the screen’s center. Yet this is not the main scope of the current paper.

In contrast to the two-choice task setting, reliable egocentric or allocentric SEs were only observed in the one-element condition. Moreover, the results showed that both, i.e., the egocentric and allocentric SEs depended on the task setup. An egocentric SE was found in the joint Go/NoGo Task setting when the task was performed alongside with a partner. This finding is well in line with existing literature on the JSE with standard stimulus setup (for review, Dolk et al., 2014) as well as the study by Ciardo et al. (2016): only with a co-actor as part of the task setup offering some sort of reference frame, an egocentric SE based on the stimulus’ position on the screen can be observed. Likewise Ciardo et al. (2016) showed that only an egocentric SE, but no allocentric SE, occurred when an enriched stimulus setup was utilized that allowed the formation of different reference frames.

Surprisingly, we also obtained an allocentric SE in the individual Go/NoGo task setting. This effect is compelling as – at outlined before – the individual Go/NoGo task setting was carried out without the partner’s involvement and thus, without a salient reference point given in order to elicit a SE. Thus, the source for the emergence of the SE can only be found in the stimulus setup itself, as the task setup *per se* did not provide any sufficiently salient reference points. At the first glance, this finding is apparently at odds with the study by Ciardo et al. (2016). Yet, although both studies used an enriched stimulus setup with using different spatial reference frames, they crucially differed in the way, how this was implemented (“external-object” vs. “same-object” approach). Therefore, these differences could explain why the stimulus setup in our study may have been salient enough in order to promote an allocentric reference frame based on the manikin’s ball position, but this might have not been the case for the vertical lines used in the other study (Ciardo et al., 2016).

Therefore, Experiment 1 showed how different spatial reference frames were shaped by the task setup. As one kind of SE was observed in the joint and individual Go/NoGo task setting (albeit being different in regard to the responsible reference frame), a salience shift between the reference frames occurred. When a partner was involved in the task as in the joint Go/NoGo task setting, the egocentric reference frame (i.e., left vs. right of the screen’s center) receives more weighting resulting in an egocentric SE for the manikin’s screen position. Contrary, without a partner in the individual Go/NoGo task setting, the allocentric reference frame became more salient capturing more details of fine-grained features of the manikins such as the side with which the manikin was holding the ball. In other words, the task setup determined whether more global features (as the spatial side of the manikin’s position, egocentric SE) or more local features (as the side of the ball, allocentric SE) of the stimulus setup were processed further in Go/NoGo task settings resulting in the formation of the corresponding reference frames. As the stimulus setup was identical for both variants of the task setup, the presence of the co-actor seemingly modulated the formation of egocentric and allocentric spatial reference frames differently. The reliance of the SE on the physical distance between the co-actors in the joint Go/NoGo task setting **(Guagnano et al., 2010)** could be used as another argument: despite identical stimulus setup, a JSE was only observed for participants within each other’s peripersonal space. With application to the current study, it could be possible that through the whole task setup (with or without a partner) a salience shift between the different spatial reference frames occurred as other details of the stimulus setup became salient depending on the individual or joint task setup. When performing the task in the individual Go/NoGo task setting, the participants focused more on the manikins itself. Contrary, when another person is seated next to the participants, the more global left/right differentiation in this scenario might be fostered resulting the salience of the egocentric reference frame. Accordingly, following this argument, the shifting between the salience of the different spatial reference frames could be the underlying principle explaining the two different SEs.

However, given the nature of this task setup, i.e., involving two participants, the shared instructions of both participants and so on, it might be possible that some carry-over effects as a function of task setup occur depending crucially on the order in which the joint or individual Go/NoGo task setting was carried out. It has been shown that carry-over effects occurred between related tasks as a spatial compatibility task (spatial location is task-relevant) and a Simon task (spatial location is task-irrelevant) (Lugli et al., 2013), even with joint and individual Go/NoGo task settings (Milanese et al., 2010). Our additional analyses with task order as a between-subjects factor partially support this idea. The additional analysis as part of the Appendix Tables displaying the allocentric SE as a function of task order might promote this idea showing that the allocentric SE was only present when the joint Go/NoGo task setting was carried out first. Yet, these values have to be interpreted with caution as they only consider half of the sample. Moreover, the possible influence of task order depending on whether the joint or individual Go/NoGo task setting was carried out first shows exactly how different task setup can potentially influence the formation of spatial reference frames. However, the potential influences of task order in our study were still clearly different from those studies observing the impact of a learning transfer between two different kinds of spatial compatibility tasks, i.e., spatial-compatibility task vs. Simon task (Milanese et al., 2010; Lugli et al., 2013). Further studies are needed in order to explicitly investigate potential task order effects between different variants of Go/NoGo Simon task settings further.

To sum up, Experiment 1 showed that the joint or individual task setup prompts the saliency of different spatial reference frames when those are directly embedded in the stimulus setup. Depending on the presence of a co-actor, either the egocentric or allocentric reference frame received more weight introducing different forms of response conflict as the source of the observed egocentric or allocentric SE. Experiment 2 tested this assumption further with different stimulus material, but otherwise identical stimulus setup and task setup.

## Experiment 2

As a salience shift between spatial reference frames triggered by the joint or individual task setup occurred in Experiment 1, this finding should be exploited further in Experiment 2. Therefore, new stimulus material was used but all other features of stimulus setup and task setup remained otherwise the same as in Experiment 1. This means, the stick-figure manikins used in Experiment 1 were quite human-like, albeit inanimate, but easily semantically connoted as such. Consequently, it might be possible that the salience shift between the different spatial reference frames was facilitated (if not enabled) by the human-like features of the manikin (e.g., body midline, two arms, two legs, head). In order to explicitly address this possibility, new abstract stimulus material was created by rearranging the parts of the stick-figure manikins in an abstract way (Figure 1B). Importantly, the abstract patterns (Experiment 2) and the manikins (Experiment 1) were physically identical; the only difference being that the abstract patterns did not represent any semantically meaningful content.

### Materials and Methods

#### Subjects

Forty-four new participants were recruited as in Experiment 1 at the University of Hildesheim (mean age: 20.89 years, 18–28 years; six male). They got partial course credit for participation. The participants mean on the IOS scale ranging from 1 to 7 (Aron et al., 1992) was 2.59 (1.48 *SD*). Three participants (mean laterality quotient = -55.00, *SD* = 42.72) were left handed (Oldfield, 1971). All participants gave written informed consent and were treated in accordance with the Declaration of Helsinki.

#### Stimuli and Apparatus

Abstract geometrical patterns were used here. They were made out of the single elements of the stick-figure manikin, but newly arranged in such a way that they did not form any meaningful object (Figure 1B). They were 26 mm in width and 35 mm in height on the screen. All other experimental details were exactly as described in Experiment 1.

#### Procedure

The procedure was identical to the one laid out in Experiment 1 except the following details. Stimuli were presented on a 17″ CRT screen. The distance of the participants to the screen was 60 cm and distance between both participants was 75 cm. The participants responded to other custom-made response buttons (without any perceivable sound associated with a button press) with their dominant hand. The responses by both participants were not covered in contrast to Experiment 1. The experiment was carried out in a different room (without separate experimental chambers). While one participant was executing the individual Go/NoGo task setting; the other participant performed another study unrelated to this experiment in the same room (yet still out of sight as separated by a black curtain).

#### Data Analysis

Errors (1.4%) and reaction time outliers (6.5%) have been removed in the same manner as in Experiment 1. The omnibus ANOVA was calculated with the within-subject factors Number of Elements (one-element condition, nine-element condition), Task Setup (joint Go/NoGo, individual Go/NoGo), egocentric Stimulus Screen Position (compatible, incompatible) to participant’s side and allocentric Stimulus Ball Position (compatible, incompatible) to participant’s side. Additional analysis was carried out including Task Order (single Go/NoGo first, joint Go/NoGo first) as between-subjects factor in the ANOVA.

### Results

**FIGURE 4:**
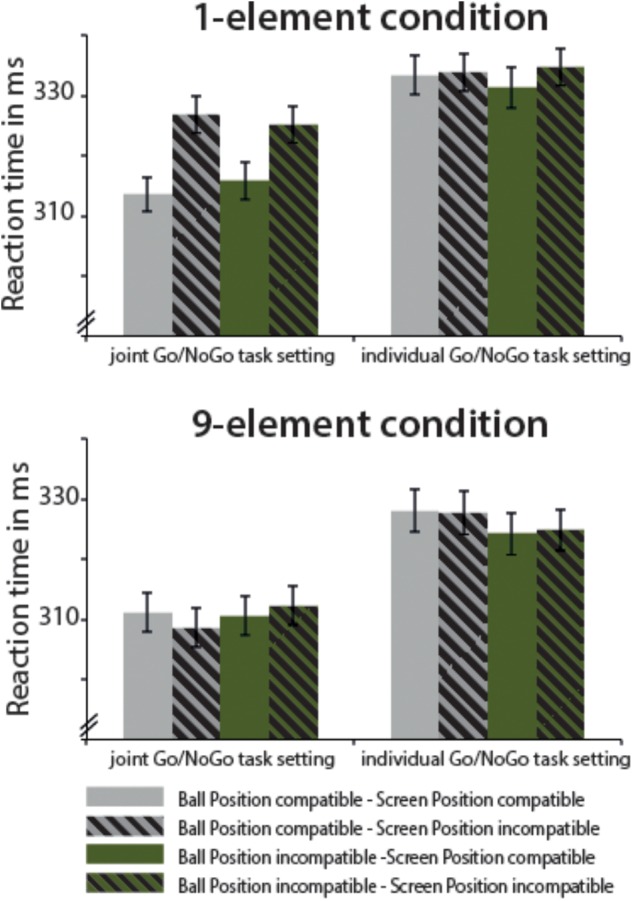
Mean reaction times and SEM for Experiment 2, separated for the one-element and the nine-element condition. Solid bars represent conditions, in which the abstract pattern’s position and the participant’s seating position are compatible (SR compatible), dashed bars display the conditions, in which the abstract pattern’s position and the participant’s seating position are incompatible (SR incompatible). Gray bars show conditions, in which the ball’s position and the participant’s seating position are compatible, green bars illustrate conditions in which the ball’s position and the participant’s seating position are incompatible. Bars are given separately for the individual and joint Go/NoGo task setting.

In the omnibus ANOVA, the main effects of Number of Elements, *F*(1,43) = 19.00, MSE = 12,445.47, *p* < 0.001, $\eta_{p}^{2}$ = 0.306 (faster responses for the nine-element condition: 318.28 ms ± 6.40, compared to the one-element condition: 326.79 ms ± 5.91) and Task Setup, *F*(1,43) = 23.15, MSE = 35,622.05, *p* < 0.001, $\eta_{p}^{2}$ = 0.350 (joint Go/NoGo task: 315.47 ms ± 6.06, single Go/NoGo task: 329.69 ms ± 6.46), were significant. Moreover, there was a main effect of egocentric Stimulus Screen Position, *F*(1,43) = 18.14, MSE = 1891.58, *p* < 0.001, $\eta_{p}^{2}$ = 0.297, pointing to faster responses for SR compatible trials (320.94 ms ± 6.09) compared to SR incompatible trials (324.22 ms ± 6.10). There were several two-way interactions, i.e., an interaction of Number of Elements and Stimulus Screen Position, *F*(1,43) = 15.90, MSE = 2012.20, *p* < 0.001, $\eta_{p}^{2}$ = 0.270, an interaction of Task Setup and Stimulus Ball Position, *F*(1,43) = 5.01, MSE = 380.97, *p* = 0.030, $\eta_{p}^{2}$ = 0.104 as well as an interaction of Task Setup and Stimulus Screen Position, *F*(1,43) = 8.27, MSE = 829.80, *p* = 0.006, $\eta_{p}^{2}$ = 0.161. Further, the three-way interaction between Number of Elements, Task Setup, and Stimulus Screen Position, *F*(1,43) = 11.65, MSE = 1070.53, *p* = 0.001, $\eta_{p}^{2}$ = 0.213, was significant (see also Figure 4). An additional ANOVA included the factor Task Order into the omnibus ANOVA (see Appendix Table A3 for all values). The interaction Task Order × Number of Elements × Task Setup × Stimulus Ball Position was significant, *F*(1,42) = 8.43, MSE = 798.20, *p* = 0.006, $\eta_{p}^{2}$ = 0.167. However, the five-way interaction was not significant.

#### One-Element Condition

In the ANOVA with the factors Task Setup, Stimulus Screen Position, and Stimulus Ball Position, the main effects of Task Setup, *F*(1,43) = 17.66, MSE = 14,813.94, *p* < 0.001, $\eta_{p}^{2}$ = 0.291 (faster responses under the joint Go/NoGo Task Setup: 320.30 ms ± 5.87 vs. the individual Go/NoGo Task Setup: 333.27 ± 6.34) and Stimulus Screen Position, *F*(1,43) = 28.03, MSE = 3902.85, *p* < 0.001, $\eta_{p}^{2}$ = 0.395 (compatible SR mapping: 323.46 ms ± 5.95 vs. incompatible SR mapping: 330.12 ms ± 5.94) reached significance. Further, there was a significant interaction between Task Setup and Stimulus Screen Position, *F*(1,43) = 20.60, MSE = 1892.67, *p* < 0.001, $\eta_{p}^{2}$ = 0.324. Additional analyses were conducted including the Task Order as between-subjects factor into the ANOVA. No main effect or interaction with Task Order was found.

##### Joint Go/NoGo task setting

In the ANOVA with the factors Stimulus Screen Position and Stimulus Ball Position, there was only a main effect of Stimulus Screen Position, *F*(1,43) = 36.25, MSE = 5615.63, *p* < 0.001, $\eta_{p}^{2}$ = 0.457. SR compatible trials were responded faster (314.65 ms ± 5.83) than SR incompatible trials (325.95 ms ± 6.07). The corresponding SEs are presented in Table 2. The additional analysis with the between-subject factor Task Order did not obtain any interaction or main effect.

**Table 2:** Mean reaction times (in ms) and standard error of the mean (SEM) for compatible and incompatible trials as a function of the mapping between Stimulus Ball Position and Stimulus Screen Position in the joint Go/NoGo Task setup and the individual Go/NoGo Task setup, respectively, as well as the egocentric and allocentric Simon Effects (SE, in ms, SEM in parenthesis), separately for the one-element and the nine-element condition, from Experiment 2.

		Joint Go/NoGo	Individual Go/NoGo
		Task setting	Task setting
One-element condition	Stimulus Ball Position compatible – Stimulus Screen Position compatible	313.55 (±5.59)	333.35 (±6.53)
	Stimulus Ball Position compatible – Stimulus Screen Position incompatible	326.79 (±6.21)	333.87 (±6.31)
	Stimulus Ball Position incompatible – Stimulus Screen Position compatible	315.75 (±6.14)	331.17 (±6.71)
	Stimulus Ball Position incompatible – Stimulus Screen Position incompatible	325.11 (±6.03)	334.70 (±6.26)
	Egocentric SE (i.e., referring to Stimulus Screen Position)	11.30 (±1.87)*	2.02 (±1.31)
	Allocentric SE (i.e., referring to Ball Position)	0.26 (±1.32)	–0.68 (±1.34)
Nine-element condition	Stimulus Ball Position compatible – Stimulus Screen Position compatible	311.11 (±6.54)	327.94 (±7.00)
	Stimulus Ball Position compatible – Stimulus Screen Position incompatible	308.61 (±6.52)	327.68 (±7.28)
	Stimulus Ball Position incompatible – Stimulus Screen Position compatible	310.57 (±6.70)	324.10 (±6.84)
	Stimulus Ball Position incompatible – Stimulus Screen Position incompatible	312.27 (±6.48)	324.75 (±6.78)
	Egocentric SE (i.e., referring to Stimulus Screen Position)	–0.40(@1.39)	0.19 (±1.56)
	Allocentric SE (i.e., referring to Ball Position)	1.56 (±1.81)	–3.38 (±1.72)*

*Asterisks refer to significant SEs (*p* ≤ 0.05).*

##### Single Go/NoGo task setting

In the corresponding ANOVA, neither a main effect nor an interaction was observed. The non-significant SEs are nonetheless given in Table 2. The additional analysis with Task Order showed an interaction between Stimulus Screen Position and Task Order, *F*(1,42) = 4.28, MSE = 303.14, *p* = 0.045, $\eta_{p}^{2}$ = 0.093 (see Appendix Table A4 for the SEs as a function of Task Order).

#### Nine-Element Condition

The ANOVA with the factors Task Setup, Stimulus Screen Position, and Stimulus Ball Position yielded a main effect of Task Setup, *F*(1,43) = 20.35, MSE = 21,084.01, *p* < 0.001, $\eta_{p}^{2}$ = 0.321, indicating faster responses under the joint Go/NoGo Task Setup (310.64 ms ± 6.41 vs. 326.12 ms ± 6.84). Further, there was a two-way interaction between Task Setup and Stimulus Ball Position, *F*(1,43) = 4.88, MSE = 537.10, *p* = 0.033, $\eta_{p}^{2}$ = 0.102. Additional analysis including the factor Task Order obtained a significant two-way interaction between Stimulus Ball Position, *F*(1,42) = 13.28, MSE = 1697.98, *p* = 0.001, $\eta_{p}^{2}$ = 0.240, as well as a three-way interaction between Task Setup, Stimulus Ball Position, and Task Order, *F*(1,42) = 8.94, MSE = 831.06, *p* = 0.005, $\eta_{p}^{2}$ = 0.175.

##### Joint Go/NoGo task setting

No main effect or interaction was obtained in a ANOVA with Stimulus Screen Position and Stimulus Ball Position. Non-significant SEs are listed in Table 2. The additional ANOVA including Task Order as a between-subject factor showed however that the factor Stimulus Ball Position was modulated by Task Order, *F*(1,42) = 27.44, MSE = 2452.43, *p* < 0.001, $\eta_{p}^{2}$ = 0.395 (see also Appendix Table A4). The allocentric SEs based on Stimulus Ball Position were significant under both Task Orders [single Go/NoGo_first: *t*(21) = 2.72, *p* = 0.013 vs. joint Go/NoGo first: *t*(21) = 4.89, *p* < 0.001], but differed in direction, resulting in an overall non-significant SE for Stimulus Ball Position.

##### Single Go/NoGo task setting

The ANOVA obtained a significant main effect of Stimulus Ball Position, *F*(1,43) = 3.86, MSE = 503.02, *p* = 0.056, $\eta_{p}^{2}$ = 0.082, indicating faster responses for SR incompatible trials compared to SR compatible trials (see also Table 2). The additional analysis with Task Order as a between-subject factor did not reveal any significant interactions involving Task Order or a main effect of Task Order.

### Discussion

Consistent with Experiment 1 and those results from the two-choice Simon task setting (Baess and Bermeitinger, unpublished), faster responses were obtained in the nine-element condition pointing to the formation of a non-spatial perceptual reference frame. Regarding our research scope, a similar result pattern was observed as in Experiment 1: an egocentric SE in the joint Go/NoGo task setting of the one-element condition and an allocentric SE in the individual Go/NoGo task setting of the nine-element condition. Again evidence was obtained for a saliency shift between different spatial reference frames (as in Experiment 1) and the non-spatial perceptual reference frame depending on the task setup. The egocentric SEs obtained in Experiments 1 and 2 were comparable in size. As the stimulus’ screen position is a rather global feature of the stimulus setup, this consistency was expected. Yet, the occurrence of the allocentric SE differed between Experiment 1 (allocentric SE in the one-element condition) and the present one (allocentric SE in the nine-element condition). Thus, what drives the distinction between the stick-figure manikins and the abstract patterns? A stick-figure manikin represents a meaningful semantic category (with well-established spatial labels, like left/right arm and so on) compared to an abstract pattern of circles and lines (without any pre-established spatial labels). Moreover, the manikins naturally introduced a differentiation between the left and right ball position. Technically, this was even introduced in the abstract geometrical patterns, but as intended, as part of a non-meaningful object. This might explain why the occurrence of the allocentric SEs was determined by the non-spatial perceptual reference frame. When one abstract pattern was presented, it might have been more difficult to form spatial codes based on the allocentric reference frame. Contrary, when a set of stimuli was presented simultaneously, it might have been easier to spot this fine-grained spatial differences required for the formation of an allocentric reference frame. Interestingly and consistent with Experiment 1, the allocentric SE was only obtained in individual Go/NoGo task setting meaning when no partner was involved in one’s own task. As the additional analysis showed, the allocentric SE in the individual Go/NoGo task setting of the nine-element condition was not influenced by task order. Therefore, carry-over effects from one task setup to the other one were less likely to be the cause of the observed salience shift between the different spatial reference frames.

To conclude, Experiment 2 showed again a salience shift between different spatial reference frames, which was modulated by the task setup and the non-spatial perceptual reference frame. The presence of a co-actor promoted the formation of an egocentric SE with regard to the abstract pattern’s screen position (and thus an egocentric reference frame). Opposite to it, when no co-actor was involved, details of the stimulus setup were focused in a much greater detail as indicated by the allocentric SE related to the ball’s position in the abstract pattern (and thus the allocentric reference frame).

## Experiment 3

Both previous experiments obtained evidence for the idea how different spatial reference frames enabled through an enriched stimulus setup are modulated by the task setup, meaning whether a Go/NoGo task setting was performed alone or together with a co-actor. The co-actor promoted the formation of spatial codes based on the egocentric reference frame in both experiments so far. In addition, without a co-actor, saying in the individual task setup, local details of the stimuli received a greater amount of processing as indicated by the formation of spatial codes based on the allocentric reference frame. Recent studies on the JSE with the standard stimulus setup have shown that it does not require a human co-actor in order to evoke a SE (for overview, Dolk et al., 2014). As reported, an external, attention-grabbing object such as a golden Japanese waving cat can also serve as a reference point crucial for the appearance of a SE (Dolk et al., 2013). Newer studies have emphasized that the stimulus modality (auditory vs. visual Simon Go/NoGo task setting) played an important factor for the efficacy of the Japanese waving cat as an attention-grabbing object (Lien et al., 2016; Puffe et al., 2017). Whereas the Japanese waving cat could successfully be used as an external salient reference point in the auditory Go/NoGo Simon task setting, it failed to do so in the visual Go/NoGo task setting. This was interpreted as evidence that the waving cat was not salient enough to induce SEs for visual stimuli. It was further assumed that the visual stimuli bound the attention more to the screen in the visual Go/NoGo task setting compared to auditory stimuli broadening the attentional focus due to the setup with loudspeakers to each side of the screen (Puffe et al., 2017). However, in these studies, visual stimuli were either presented centrally superimposed on a task-irrelevant directional photo of a hand (Lien et al., 2016) or spatially aligned left or right from the midline of the screen (Puffe et al., 2017). In both studies, no SE could be obtained for visual Go/NoGo task settings, neither in the condition with the Japanese waving cat nor without. Yet, Stenzel and Liepelt (2015) provided evidence for SEs in an individual visual Go/NoGo task setting when a photo of a Japanese waving cat was displayed in one corner of the screen, as part of the task setup^1^, but clearly outside of the critical stimulus. Under this task setup, reliable SEs were obtained for both, a photo of a human hand or a photo of the Japanese waving cat. Interestingly, the size of the SE did not vary between a photo of the Japanese waving cat or a human hand. This illustrates the feasibility to induce spatial codes also under a visual Go/NoGo task setting if the task setup is salient enough to include spatial reference points.

As attention-grabbing object such as the Japanese waving cat are in principal salient enough to support the formation of spatial codes as the source of the SE (cf. Dolk et al., 2013), the present study aimed at replicating Experiment 1 by replacing the co-actor with a Japanese waving cat. This manipulation allowed us to investigate the formation of spatial reference frames within the enriched stimulus setup as used in Experiments 1 and 2 in a Go/NoGo task setting without any co-actor. Because the task setup never included a co-actor, Experiment 3 provides some kind of baseline of how different spatial reference frames could be formed in individual Go/NoGo task settings without the influence of a co-actor, but with or without the potential impact of an external, attention-grabbing object.

### Materials and Methods

#### Subjects

Forty-one new participants were recruited for this study. One participant was excluded due to lack of compliance (two-choice responses in the individual Go/NoGo task setting). One further participant was not naïve to the purpose of the study due to attending a course by one of the authors. Thus, the final sample consisted of 39 participants (mean age: 22.0 years, 18–35 years; five male). Three participants (laterality quotient: -55.00, *SD* = 44.44) were left handed according to a handedness questionnaire (Oldfield, 1971). One participant did not have a preferred hand. One participant did not fill out the handedness questionnaire and questions regarding its age and gender. All participants gave written informed consent and were treated in accordance with the Declaration of Helsinki.

#### Stimuli and Apparatus

The same stick-figure manikins as in Experiment 1 on a 16″ CRT-monitor were used. In contrast to previous experiments, this experiment was carried out alone without a co-actor’s involvement. For the sake of consistency, two chairs were placed in front of the monitor, although the second chair was never used (distance between both chairs: 5 cm). Further, only the one-element condition was executed under two variations of the task setup. In the cat-present task setup, a golden Japanese waving cat (height: 17 cm; width: 10.5 cm, depth: 7 cm) was placed left side of the monitor and participant (Figure 1C). The automatic battery-driven movement of its left arm produced a barely noticeable, unsystematic waving sound as part of the waving movement. The participants were clearly able to see the cat in their peripheral visual field. In the cat-absent task setup, the whole arrangement remained the same except that the cat was not visible any more (it was hidden inside of a paper cylinder) and was switched off. The participants performed under both task setups as an individual Go/NoGo task setting.

#### Procedure

The order of the cat-present and cat-absent task setups was counterbalanced across the participants. In order to make the necessary changes in the testing chambers, the participants were briefly asked to leave the testing chamber with the explanation that the experimental leader had to start the new condition. The experiment instructors changed the task setup in the test chambers according to the counterbalanced order. As indicated by Figure 1C, the two test chambers were yet other ones than used so far. Importantly, the cat itself never left the test chamber but was hidden inside of a paper cylinder (not visible for the participants) in the cat-absent task setup. In the cat-present task setup, the cat was placed before the paper cylinder. To maintain symmetry, a lamp was positioned on the right side of the monitor, which remained switched on during the whole experiment. All participants were seated on the right chair and used the custom-made response button of Experiment 2 to react with their right index finger (distance monitor and participant: 52, respectively, 55 cm depending on the test chamber). Only one response button was placed on the table. The participant sat throughout the experiment on the right chair and the cat (if present) was always at the left side of the screen. Half of the participants responded to blue stimuli and the other half to yellow stimuli.

#### Data Analysis

Errors (0.42%) and reaction time outliers (2.87%) were identified as in previous experiments. The omnibus ANOVA was calculated with the within-subject factors Task Setup (cat-present, cat-absent), egocentric Stimulus Screen Position (compatible, incompatible), and allocentric Stimulus Ball Position (compatible, incompatible).

### Results

**FIGURE 5:**
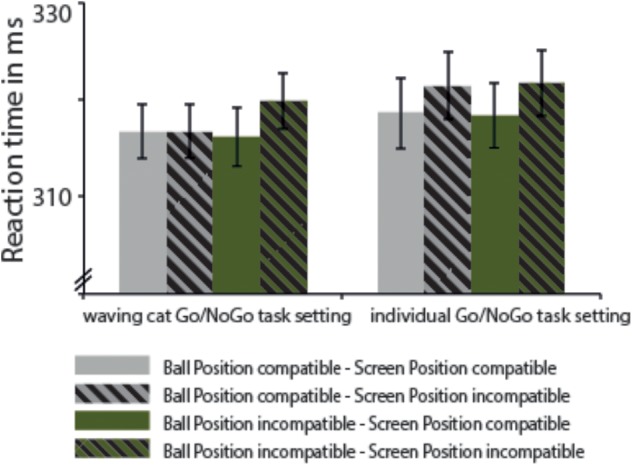
Mean reaction times and SEM for Experiment 3. Solid bars represent conditions in which the manikin’s position and the participant’s seating position are compatible (SR compatible), dashed bars display conditions in which the manikin’s position and participant’s seating position are incompatible (SR incompatible). Gray bars show conditions in which the ball’s position and the participant’s seating position are compatible, green bars illustrate conditions in which the ball’s position and the participant’s seating position are incompatible. Bars are given separately for the individual Go/NoGo task setting with and without Japanese waving cat.

In the omnibus ANOVA, a main effect of egocentric Stimulus Screen Position was obtained, *F*(1,38) = 6.28, MSE = 469.94, *p* = 0.017, $\eta_{p}^{2}$ = 0.142, 90% CI of the effect size [0.01; 0.31], indicating faster responses for SR compatible trials (317.48 ms ± 6.21) compared to SR incompatible ones (319.93 ms ± 6.08), irrespective of the task setup. The corresponding egocentric SE was 2.45 ms (±0.98). The other main effects or the interactions were clearly not significant (all *p*s > 0.3), see also Table 3 and Figure 5.

**Table 3:** Egocentric and allocentric SE from Experiment 3.

	Egocentric SE	Allocentric SE
	(i.e., referring to	(i.e., referring to
	Stimulus Screen	Ball Position) in
	Position) in	milliseconds
	millisecond (SEM)	(SEM)
Individual Go/NoGo Task setting with Japanese waving cat	1.84 (±1.15)	1.30 (±1.48)
Individual Go/NoGo Task setting without Japanese waving cat	3.07 (±1.58)	0.03 (±1.23)

### Discussion

Experiment 3 obtained evidence for an egocentric SE when using the stimulus setup with different possibilities to form spatial codes in an individual Go/NoGo task setting. The egocentric SE was completely unaffected by the presence of a Japanese waving cat. At the first glance, this result is surprising as previous research failed to report a reliable SEs in a visual Go/NoGo task setting (e.g., Hommel, 1996). Yet, the emergence of an egocentric SE in our study might as well illustrate that spatial reference frames may be utilized also in Go/NoGo Simon task settings provided that sufficiently salient reference points were embedded in the stimulus setup. This might explain why we found a small but reliable egocentric SE when other’s failed to do so. This notion is further supported by the fact even changing the task setup by including the Japanese waving cat did not modulate the SEs. Therefore, one might even claim that the Japanese waving cat in our task setup did not serve as a spatial reference point as in other studies (Dolk et al., 2013; Lien et al., 2016; Puffe et al., 2017). The stimulus setup used in our experiments with the possibility to form different spatial reference frames was already salient enough to promote the formation of the egocentric reference frame. In line with this statement, the Japanese waving cat did not add “new” reference points to the task setup, on top of the ones already inherent in the stimulus setup of our study. Thus, our overall egocentric SE in both individual Go/NoGo task settings might illustrate that our stimulus setup is *per se* salient enough to boost the formation of spatial reference frames. Finally, one might wonder why no allocentric SE occurred at all in Experiment 3. Following our previous experiments, an allocentric SE consistently occurred in an individual Go/NoGo task setting, but not in a joint Go/NoGo task setting. Hence, the allocentric SE in our previous studies was demonstrated when the whole task setup involved a human co-actor. Only under this condition, we observed a salience shift between more global features of the stimuli (as indicated by the egocentric SE) and more local features of the stimuli (as evident by the allocentric SE).

To conclude, this study showed that the stimulus setup itself could promote the formation of spatial reference frames. Other, external attention-grabbing objects did not modulate the spatial reference frames further.

## General Discussion

The present study examined how the formation of different egocentric and allocentric reference frames was modulated by the task setup, performing a visual Go/NoGo Simon task setting alone or together with a co-actor. Central to our studies was the usage of an enriched stimulus setup (“same-object-approach”) allowing the simultaneous formation of egocentric, allocentric, and even a non-spatial perceptual reference frame. The possibility that the stimulus setup itself might include enough salient reference points in order to establish cognitive conflict as the source of the SE has not yet received much attention in Go/NoGo Simon task setting. Experiment 1 gave evidence for an egocentric SE under joint Go/NoGo task setting and an allocentric SE under individual Go/NoGo task setting. Both SEs were obtained when one critical stimulus was presented on the screen (one-element condition). Experiment 2 confirmed, in principal, previous results using abstract stimulus material. Here, an egocentric SE was obtained in the joint Go/NoGo task setting when one stimulus was shown on the screen and an allocentric SE was found in the individual Go/NoGo task setting when a set of nine identical stimuli were shown on the screen allowing the formation of a non-spatial perceptual reference frame by applying the Gestalt principle of grouping. Lastly, Experiment 3 investigated whether an external, attention-grabbing object such as the Japanese waving cat would also offer additional reference points (besides the ones already inherent in our stimulus setup) in the task setup. The finding of an overall egocentric SE totally independent of the Japanese waving cat showed that our enriched stimulus setup is already salient enough to provide reference points as a core of spatial conflict. The reference points offered by the Japanese waving cat did not add anything additionally to the scenario. In the following, we will discuss our results along these two main lines, i.e., (i) the salience shift between egocentric and allocentric reference frames and (ii) the influence of stimulus setup and task setup on the formation of spatial reference frames.

### Salience Shift Between Egocentric and Allocentric Reference Frames: The Influence of Task Setup

When the participants worked at any point during the experimental session together with a human co-actor (as in Experiments 1 and 2), we observed an egocentric SE in the joint Go/NoGo task setting and even an allocentric SE in the individual Go/NoGo task setting, albeit no co-actor or other attention-grabbing object was involved in the task setup. This salience shift between spatial reference frames (egocentric vs. allocentric) as a function of individual or joint task setup is compelling. Previous work showed that a JSE emerged in a Go/NoGo task setting when a human co-actor or an external, attention-grabbing object was present to provide spatial reference crucial for the appearance of a SE (for overview, Dolk et al., 2014). Both, the human co-actor or the external, attention-grabbing object enriched the joint task setup as a part of the representation of the whole task. In these studies, the crucial comparison between joint and individual task setups illustrated how a co-actor or an external object could be used as salient reference frames. These studies utilized a stimulus setup that allowed only one possible spatial reference frame, namely the egocentric reference frame based on the stimulus’ screen position. Yet, the possibility that the stimulus setup itself could foster the formation of spatial codes provided its sufficient salience has so far not yet been systematically considered. Only the study by Ciardo et al. (2016) used some sort of enriched stimulus setup (“external-object-approach”) while varying the task setup. Here, an egocentric SE (also labeled SE for hemispace) was reported in the joint Go/NoGo task setting, but – in contrast to our variation of enriched stimulus setup (“same-object-approach”) – no allocentric SE (also labeled as relative position within each hemispace). This discrepancies in the allocentric reference frame might be explained best by recalling the differences in the enriched stimulus setups used in their and our study: while in our study the reference points for the allocentric reference frame were in close proximity (or even part of more global features) of the critical stimulus, the reference points for the allocentric reference frame were in the other study clearly separated from the critical stimulus. It has been shown elsewhere that the enriched stimulus setup used in our study is *per se* salient enough to simultaneously provoke different spatial and even non-spatial perceptual reference frames (Baess and Bermeitinger, unpublished). This was not observed in the study Ciardo et al. (2016) using a different approach to enrich the stimulus setup (see also for the two-choice task setting, Roswarski and Proctor, 1996). We might therefore conclude that a salience shift between different spatial reference frames occurred in our studies depending on the task setup: When the Go/NoGo task setting involved a co-actor, the global features of the stimulus setup (i.e., the spatial location of the stimulus with regard to its position on the screen) received detailed processing resulting in an egocentric SE. Contrary, when no co-actor was part of the task setup as in the individual Go/NoGo task setting, more local features of the stimulus setup were elaborated leading to the emergence of an allocentric SE. This idea of a salience shift between different spatial reference frames in a Go/NoGo Simon task setting has so far not yet been shown. Most likely, the previously used (enriched or standard) stimulus setup was not salient enough in order to foster cognitive conflict based on different spatial reference systems. This salience shift between egocentric and allocentric reference frames could follow the idea of an intentional weighting mechanism suggested as a central principle underlying human cognitive control (cf. Memelink and Hommel, 2013). In a nutshell, some features (e.g., the left/right labels with regard to the screen’s center) are weighted more strongly during the joint task setup as a co-actor is next to the corresponding agent, so that the left/right features representing an egocentric reference frame received a stronger emphasis resulting in an egocentric SE for the joint Go/NoGo task setting. In contrast, when the very same task is executed alone, those features promoting an egocentric reference frame might under this condition be less salient and received less weight. Alternatively, the fine-grained local features of the manikin itself, i.e., the side of the “hand” holding the ball, might now receive more weight leading to the dominance of an allocentric reference frame. To state, the observed salience shifts between different spatial reference frames show how a co-actor’s presence can change the relevance of reference frames within the same enriched stimulus setup.

The human flexibility to adopt between different reference frames and even perspectives has been shown in other paradigms as well. Samson et al. (2010) showed that the perspective of a human avatar influenced one’s own perspective (“altercentric intrusions”) in visual perspective taking experiments, although the participants were explicitly instructed not to do so. Here, the perspective of the avatar could not easily be ignored. In this study, participants had to mentally rotate themselves into the avatar’s position in order to take over the perspective of the avatar. However, the participants in our study were neither instructed to explicitly take over a certain perspective nor did the stick-figure manikin’s frontal view promote the idea of mentally rotating oneself into the manikin’s perspective. However, the human automatic ability to mentally take over other’s perspective might work as an explanation for the differences in the allocentric reference frame between the stick-figure manikins and the abstract geometrical patterns.

A study by Freundlieb et al. (2017) illustrated that spatial compatibility effects as a marker of visuospatial perspective taking occurred only when the co-actor had visual access to the stimulus setup, even when the co-actor performed a different task. As the co-actors in our joint Go/NoGo task setting performed the same Simon task with mutual visual access, this might illustrate further why the egocentric reference frame might be the dominant one. It has also been show in a Navon-Task that the reaction times slowed down when different features of the same stimulus (global vs. local) had to be considered within a pair of co-actors (Bockler et al., 2012). When the co-actors focus of attention (e.g., global features) differed from one’s own focus of attention (e.g., local features), this led to a conflict in selecting the appropriate response as evident by a slowdown of response times. In our study, the switch between global and local features of the stimulus setup took place uninstructed and automatically when the task setup changed between individual and joint Go/NoGo task settings.

### The Influence of Stimulus Setup and Task Setup on the Formation of Spatial Reference Frames

Our Experiment 3 illustrated the influence of the overall task setup as the practical abstraction level of “task shaping” (Prinz, 2015; Dolk and Prinz, 2016). When no “task shaping” could take place in any form in the task setup, i.e., no co-actor was at any point involved in the task setup, the enriched stimulus setup of our study evoked the formation of spatial reference frames differently. Here, only an overall egocentric SE was yielded, unrelated to other attention-grabbing objects as part of the task setup. As the stimulus setup utilized in Experiments 1 and 3 was identical and the presence of a co-actor being the only difference, this might illustrate the “core” impact of a task setup involving a co-actor. In other words, when the overall task setup (i.e., the experiment in general) involved a co-actor, even independent of the current task setup (i.e., the joint or individual task setup), this might be salient enough to represent – in some extent – the co-actor as part of the overall task setup. Yet, the level of co-actor’s representation as part of the overall task setup might be a rather general one, for example, it could be restricted to acknowledging that the overall task setup involved, at some point, a human co-actor. Hence, this level of “joint encounter” as part of the overall task setup, inseparably inherent within this line of research, could possibly boost the mechanisms assigning different weights to different spatial reference frames during the processing the identical stimulus setup under different Go/NoGo Task setups. Consequently, the effects were salient schifts between egocentric and allocentric reference points as observed between joint and individual Go/NoGo task settings. These salience shifts did not occur when no co-actor, but an external attention-grabbing object, was part of the task setup. Thus, the effects of the human co-actor were seemingly two-folded: (i) the co-actor’s presence shapes the overall task setup and (ii) the-co-actor’s presence reinforces the egocentric reference frame in the joint task setup. Importantly, as Experiment 3 showed, the co-actor was not *per se* required for the formation of spatial codes within the egocentric reference frame, but served as a trigger (i.e., weight) in order to foster the switch between different spatial reference frames across different task setups.

## Conclusion

Our series of experiments provides evidence how an enriched stimulus setup influenced the formation of spatial, i.e., egocentric and allocentric reference frames differently for the joint and individual task setup. SEs were obtained in Go/NoGo task settings when using a stimulus setup that provided sufficient reference points for the formation of spatial reference frames. If the overall task setups involving at some point a co-actor, a salience shift between spatial reference frames and thus between global and local details of the stimulus setup as the source of the underlying cognitive conflict was observed. Further studies are required in order to scrutinize the interplay between stimulus setup and task setup in social and non-social contexts more thoroughly.

## Ethics Statement

This study was carried out in accordance with the recommendations of the German Society of Psychology’s research standards. The protocol was approved by the local ethic committee (“Fachbereich 1”) of the University of Hildesheim. All subjects gave written informed consent in accordance with the Declaration of Helsinki.

## Author Contributions

PB designed, programmed, and analyzed all experiments and wrote the manuscript. TW collected the data for Experiment 1 as part of his Bachelor’s degree. CB developed the initial version of the paradigm and commented on parts of the manuscript.

## Conflict of Interest Statement

The authors declare that the research was conducted in the absence of any commercial or financial relationships that could be construed as a potential conflict of interest.

## Footnotes

1 As the photo of the cat was not part of relevant stimulus setup, we consider it a modulation of task setup in a reading that the features of an enriched task setup were projected on the screen itself instead of spatially aligned in the scenario.
