# Supplementary material for: Sharing Different Reference Frames: How Stimulus Setup and Task Setup Shape Egocentric and Allocentric Simon Effects
Source: Front Psychol. 2018 Nov 30;9:2063. doi: 10.3389/fpsyg.2018.02063 (PMC6284048; doi:10.3389/fpsyg.2018.02063)
Supplement: Supplementary file 1 [file Table_1.pdf]

TABLE A1 | A summary table for the Omnibus ANOVA on the mean reaction times as a function of the between-subject factor Task Order (single Go/NoGo task first – joint Go/NoGo task second vs. joint Go/NoGo task first – single Go/NoGo task second) and the within-subject factors Number of Element (one-element condition, nine-element condition), Task Setup (joint Go/NoGo, single Go/NoGo), Stimulus Screen Position (compatible, incompatible), Stimulus Ball Position (compatible, incompatible) in Experiment 1.

Effect	*df*	*F*	*p*	η_p^2
Task Order	1,37	<1	0.599	0.008
Number of Element	1,37	22.42	<0.001	0.377
Task Setup	1,37	<1	0.396	0.020
Stimulus Ball Position	1,37	3.16	0.084	0.079
Stimulus Screen Position	1,37	3.71	0.062	0.091
Task Order × Number of Element	1,37	<1	0.869	0.001
Task Order × Task Setup	1,37	<1	0.853	0.001
Task Order × Stimulus Ball Position	1,37	13.85	0.001	0.272
Task Order × Stimulus Screen Position	1,37	<1	0.777	0.002
Number of Element × Task Setup	1,37	<1	0.506	0.012
Number of Element × Stimulus Ball Position	1,37	<1	0.353	0.023
Number of Element × Stimulus Screen Position	1,37	3.93	0.055	0.096
Task Order × Number of Element × Task Setup	1,37	<1	0.848	0.001
Task Order × Number of Element × Stimulus Ball Position	1,37	<1	0.496	0.013
Task Order × Number of Element × Stimulus Screen Position	1,37	<1	0.714	0.004
Task Setup × Stimulus Ball Position	1,37	<1	0.430	0.017
Task Setup × Stimulus Screen Position	1,37	2.48	0.124	0.063
Task Order × Task Setup × Stimulus Ball Position	1,37	<1	0.592	0.008
Task Order × Task Setup × Stimulus Screen Position	1,37	<1	0.594	0.008
Stimulus Ball Position × Stimulus Screen Position	1,37	1.70	0.201	0.044
Task Order × Stimulus Ball Position × Stimulus Screen Position	1,37	<1	0.674	0.005
Number of Element × Task Setup × Stimulus Ball Position	1,37	4.91	0.033	0.117
Number of Element × Task Setup × Stimulus Screen Position	1,37	2.29	0.139	0.058
Number of Element × Stimulus Ball Position × Stimulus Screen Position	1,37	1.51	0.226	0.039
Task Order × Number of Element × Task Setup × Stimulus Ball Position	1,37	3.21	0.081	0.080
Task Order × Number of Element × Task Setup × Stimulus Screen Position	1,37	4.62	0.038	0.111
Task Order × Number of Element × Stimulus Ball Position × Stimulus Screen Position	1,37	<1	0.351	0.024
Task Setup × Stimulus Ball Position × Stimulus Screen Position	1,37	<1	0.396	0.020
Task Order × Task Setup × Stimulus Ball Position × Stimulus Screen Position	1,37	2.56	0.118	0.065
Number of Element × Task Setup × Stimulus Ball Position × Stimulus Screen Position	1,37	2.35	0.133	0.060
Task Order × Number of Element × Task Setup × Stimulus Ball Position × Stimulus Screen Position	1,37	<1	0.804	0.002
