# Supplementary material for: Sharing Different Reference Frames: How Stimulus Setup and Task Setup Shape Egocentric and Allocentric Simon Effects
Source: Front Psychol. 2018 Nov 30;9:2063. doi: 10.3389/fpsyg.2018.02063 (PMC6284048; doi:10.3389/fpsyg.2018.02063)
Supplement: Supplementary file 2 [file Table_2.pdf]

TABLE A2 | Mean reaction times (in ms) and SEM as a function of Task Order (single Go/NoGo first – joint Go/NoGo second; joint Go/NoGo first – single Go/NoGo second), Task Setup (joint Go/NoGo, single Go/NoGo), Stimulus Ball Position (compatible, incompatible), and Stimulus Screen Position (compatible, incompatible), as well as the egocentric and allocentric Simon Effects (SE, in ms, SEM in parenthesis), for the one-element condition from Experiment 1.

		**Joint Go/NoGo Task setting**	**Individual Go/NoGo Task setting**
Single Go/NoGo first (*N* = 20)	Stimulus Ball Position compatible – Stimulus Screen Position compatible	368.59(±10.94)	380.77(±13.15)
	Stimulus Ball Position compatible – Stimulus Screen Position incompatible	387.24(±12.88)	374.28(±12.51)
	Stimulus Ball Position incompatible – Stimulus Screen Position compatible	372.98(±12.93)	375.93(±13.40)
	Stimulus Ball Position incompatible – Stimulus Screen Position incompatible	377.59(±10.38)	377.95(±12.42)
	Egocentric SE (i.e., referring to Stimulus Screen Position)	11.63(±3.08)	−2.23(±2.51)
	Allocentric SE (i.e., referring to Ball Position)	−2.63(±3.31)	−0.58(±1.86)
Joint Go/NoGo first (*N* = 19)	Stimulus Ball Position compatible – Stimulus Screen Position compatible	380.22(±12.70)	377.12(±11.64)
	Stimulus Ball Position compatible – Stimulus Screen Position incompatible	387.42(±10.87)	380.29(±13.71)
	Stimulus Ball Position incompatible – Stimulus Screen Position compatible	379.83(±11.97)	390.32(±13.71)
	Stimulus Ball Position incompatible – Stimulus Screen Position incompatible	386.92(±11.59)	399.14(±14.47)
	Egocentric SE (i.e., referring to Stimulus Screen Position)	7.15(±5.99)	6.00(±3.50)
	Allocentric SE (i.e., referring to Ball Position)	−0.45(±4.30)	16.03(±5.26)
