# Supplementary material for: Sharing Different Reference Frames: How Stimulus Setup and Task Setup Shape Egocentric and Allocentric Simon Effects
Source: Front Psychol. 2018 Nov 30;9:2063. doi: 10.3389/fpsyg.2018.02063 (PMC6284048; doi:10.3389/fpsyg.2018.02063)
Supplement: Supplementary file 3 [file Table_3.pdf]

TABLE A3 | A summary table for the Omnibus ANOVA on the mean reaction times as a function of the between-subjects factor Task Order (single Go/NoGo task first – joint Go/NoGo task second vs. joint Go/NoGo task first – single Go/NoGo task second) and the within-subject factors Number of Element (one-element condition, nine-element condition), Task Setup (joint Go/NoGo, single Go/NoGo), Stimulus Screen Position (compatible, incompatible), Stimulus Ball Position (compatible, incompatible) in Experiment 2.

Effect	*df*	*F*	*p*	η_p^2
Task Order	1,42	<1	0.707	0.003
Number of Element	1,42	20.37	<0.001	0.327
Task Setup	1,42	23.44	<0.001	0.358
Stimulus Ball Position	1,42	<1	0.493	0.011
Stimulus Screen Position	1,42	18.06	<0.001	0.301
Task Order × Number of Element	1,42	4.11	0.049	0.089
Task Order × Task Setup	1,42	1.54	0.221	0.035
Task Order × Stimulus Ball Position	1,42	8.01	0.007	0.160
Task Order × Stimulus Screen Position	1,42	<1	0.374	0.019
Number of Element × Task Setup	1,42	<1	0.370	0.019
Number of Element × Stimulus Ball Position	1,42	<1	0.630	0.006
Number of Element × Stimulus Screen Position	1,42	15.54	<0.001	0.270
Task Order × Number of Element × Task Setup	1,42	<1	0.338	0.022
Task Order × Number of Element × Stimulus Ball Position	1,42	8.43	0.006	0.167
Task Order × Number of Element × Stimulus Screen Position	1,42	<1	0.844	0.001
Task Setup × Stimulus Ball Position	1,42	5.14	0.029	0.109
Task Setup × Stimulus Screen Position	1,42	8.27	0.006	0.165
Task Order × Task Setup × Stimulus Ball Position	1,42	2.11	0.153	0.048
Task Order × Task Setup × Stimulus Screen Position	1,42	1.00	0.322	0.023
Stimulus Ball Position × Stimulus Screen Position	1,42	<1	0.504	0.011
Task Order × Stimulus Ball Position × Stimulus Screen Position	1,42	<1	0.380	0.018
Number of Element × Task Setup × Stimulus Ball Position	1,42	1.86	0.180	0.042
Number of Element × Task Setup × Stimulus Screen Position	1,42	11.91	0.001	0.221
Number of Element × Stimulus Ball Position × Stimulus Screen Position	1,42	1.19	0.283	0.027
Task Order × Number of Element × Task Setup × Stimulus Ball Position	1,42	8.43	0.006	0.167
Task Order × Number of Element × Task Setup × Stimulus Screen Position	1,42	1.97	<1	0.045
Task Order × Number of Element × Stimulus Ball Position × Stimulus Screen Position	1,42	<1	0.416	0.016
Task Setup × Stimulus Ball Position × Stimulus Screen Position	1,42	<1	0.510	0.010
Task Order × Task Setup × Stimulus Ball Position × Stimulus Screen Position	1,42	1.28	0.264	0.030
Number of Element × Task Setup × Stimulus Ball Position × Stimulus Screen Position	1,42	2.65	0.149	0.049
Task Order × Number of Element × Task Setup × Stimulus Ball Position × Stimulus Screen Position	1,42	<1	0.501	0.011
