# Supplementary material for: Sharing Different Reference Frames: How Stimulus Setup and Task Setup Shape Egocentric and Allocentric Simon Effects
Source: Front Psychol. 2018 Nov 30;9:2063. doi: 10.3389/fpsyg.2018.02063 (PMC6284048; doi:10.3389/fpsyg.2018.02063)
Supplement: Supplementary file 4 [file Table_4.pdf]

**TABLE A4 |** Mean reaction times (in ms) and SEM as a function of Task Order (single Go/NoGo first – joint Go/NoGo second; joint Go/NoGo first – single Go/NoGo second), Task setup (joint Go/NoGo, single Go/NoGo), Stimulus Ball Position (compatible, incompatible), and Stimulus Screen Position (compatible, incompatible) for the one-element condition and nine-element condition, as well as the egocentric and allocentric Simon Effects (SE, in ms, SEM in parenthesis), from Experiment 2.

| | | | **Joint Go/NoGo Task setting** | **Individual Go/NoGo Task setting** |
|---|---|---|---|---|
| One-element condition | Single Go/NoGo first (*N* = 22) | Stimulus Ball Position compatible – Stimulus Screen Position compatible | 312.04 (±8.55) | 336.93 (±9.79) |
| | | Stimulus Ball Position compatible – Stimulus Screen Position incompatible | 325.87 (±9.26) | 334.87 (±8.80) |
| | | Stimulus Ball Position incompatible – Stimulus Screen Position compatible | 314.94 (±9.51) | 333.41 (±9.63) |
| | | Stimulus Ball Position incompatible – Stimulus Screen Position incompatible | 325.49 (±9.32) | 334.27 (±9.25) |
| | | Egocentric SE (i.e., referring to Stimulus Screen Position) | 12.19 (±2.76) | −0.60 (±2.04) |
| | | Allocentric SE (i.e., referring to Ball Position) | 1.26 (±1.93) | −2.06 (±1.72) |
| | Joint Go/NoGo first (*N* = 22) | Stimulus Ball Position compatible – Stimulus Screen Position compatible | 315.05 (±7.70) | 329.78 (±8.81) |
| | | Stimulus Ball Position compatible – Stimulus Screen Position incompatible | 327.70 (±8.49) | 332.88 (±9.24) |
| | | Stimulus Ball Position incompatible – Stimulus Screen Position compatible | 316.57 (±7.98) | 328.93 (±9.55) |
| | | Stimulus Ball Position incompatible – Stimulus Screen Position incompatible | 324.72 (±7.86) | 335.12 (±8.65) |
| | | Egocentric SE (i.e., referring to Stimulus Screen Position) | 10.40 (±2.59) | 4.65 (±1.50) |
| | | Allocentric SE (i.e., referring to Ball Position) | −0.73 (±1.81) | 0.70 (±2.04) |
| Nine-element condition | Single Go/NoGo first | Stimulus Ball Position compatible – Stimulus Screen Position compatible | 318.25 (±10.68) | 335.40 (±10.75) |
| | | Stimulus Ball Position compatible – Stimulus Screen Position incompatible | 312.37 (±10.86) | 334.96 (±10.54) |
| | | Stimulus Ball Position incompatible – Stimulus Screen Position compatible | 307.63 (±10.74) | 330.34 (±10.66) |
| | | Stimulus Ball Position incompatible – Stimulus Screen Position incompatible | 311.18 (±10.71) | 330.62 (±10.55) |
| | | Egocentric SE (i.e., referring to Stimulus Screen Position) | −1.17 (±2.07) | −0.08 (±2.26) |
| | | Allocentric SE (i.e., referring to Ball Position) | −5.91 (±2.17) | −4.70 (±2.54) |
| | Joint Go/NoGo first | Stimulus Ball Position compatible – Stimulus Screen Position compatible | 303.96 (±7.49) | 320.47 (±8.94) |
| | | Stimulus Ball Position compatible – Stimulus Screen Position incompatible | 304.85 (±7.40) | 320.38 (±10.03) |
| | | Stimulus Ball Position incompatible – Stimulus Screen Position compatible | 313.50 (±8.22) | 317.87 (±8.63) |
| | | Stimulus Ball Position incompatible – Stimulus Screen Position incompatible | 313.36 (±7.57) | 318.88 (±8.57) |
| | | Egocentric SE (i.e., referring to Stimulus Screen Position) | 0.37 (±1.89) | 0.47 (±2.20) |
| | | Allocentric SE (i.e., referring to Ball Position) | 9.03 (±1.85) | −2.06 (±2.34) |
